# Niche conservatism of *Aedes albopictus* and *Aedes aegypti* - two mosquito species with different invasion histories

**DOI:** 10.1038/s41598-018-26092-2

**Published:** 2018-05-16

**Authors:** Sarah Cunze, Judith Kochmann, Lisa K. Koch, Sven Klimpel

**Affiliations:** 1 Goethe-University, Institute of Ecology, Evolution and Diversity; Max-von-Laue-Str. 13, D-60438 Frankfurt/ M., Germany; 2 Senckenberg Gesellschaft für Naturforschung, Senckenberg Biodiversity and Climate Research Centre; Senckenberganlage 25, D-60325 Frankfurt/ M., Germany

## Abstract

Biological invasions have been associated with niche changes; however, their occurrence is still debated. We assess whether climatic niches between native and non-native ranges have changed during the invasion process using two globally spread mosquitoes as model species, *Aedes albopictus* and *Aedes aegypti*. Considering the different time spans since their invasions (>300 vs. 30–40 years), niche changes were expected to be more likely for *Ae*. *aegypti* than for *Ae*. *albopictus*. We used temperature and precipitation variables as descriptors for the realized climatic niches and different niche metrics to detect niche dynamics in the native and non-native ranges. High niche stability, therefore, no niche expansion but niche conservatism was revealed for both species. High niche unfilling for *Ae*. *albopictus* indicates a great potential for further expansion. Highest niche occupancies in non-native ranges occurred either under more temperate (North America, Europe) or tropical conditions (South America, Africa). *Aedes aegypti* has been able to fill its native climatic niche in the non-native ranges, with very low unfilling. Our results challenge the assumption of rapid evolutionary change of climatic niches as a requirement for global invasions but support the use of native range-based niche models to project future invasion risk on a large scale.

## Introduction

Ongoing change in climatic conditions is expected to influence species’ distributions, which in turn will affect biodiversity patterns^1,2^. Biological invasions are promoted by climate change^3^ and are further enhanced by increasing global trade and tourism. More recently, they have also been associated with changes in the species’ realized climatic niches, with reports of niche changes between native and non-native populations^4,5^. However, the evidence of such niche changes during invasion processes is currently under debate^6,7^ and might be species-specific: whereas several authors argue for a climatic niche shift of different plant species in the invaded range^8–10^, a study by Petitpierre *et al*.^11^ suggests that climatic niches did not change substantially for most of the 50 investigated invasive terrestrial plant species. Strubbe *et al*.^12^ confirmed niche conservatism for non-native birds in Europe and Strubbe *et al*.^5^ proposed conserved niches in the introduced ranges (either Europe or North America) for most of the 29 vertebrate species studied.

Generally, climatic niche shifts contradict the assumption of a niche conservatism, which implies that species retain their niches in space and time^13^. Ecological niche modelling, which is the most commonly used approach to assess, firstly, the impact of climate change on biodiversity and secondly, invasion risk, strongly relies on this assumption. One way to assess whether the species’ niche is conserved over time and space is to investigate the distribution in the niche space (as an estimation of species’ realized niche) focusing on native and non-native ranges of the species. The investigation of species’ niches can thus be used to better assess and improve ecological niche modelling results (e.g. Aguirre-Gutiérrez *et al*.^14^). Furthermore, comparisons between species’ native and non-native range climatic niches may identify species that have undergone adaptive evolutionary changes during the invasion process (e.g. change of the fundamental climatic niche), but might generally benefit a better understanding of different niche dynamics^6^. Drivers other than climate can also be involved in niche shifts during invasions, e.g. ecological drivers such as biotic interactions. These should ideally not be considered separately^15^. However, data are usually available only on a different, much smaller scale and can therefore not be incorporated in the same models.

Here, *Aedes albopictus* and *Aedes aegypti*, two mosquito species that are listed among the world’s worst invasive alien species (Global Invasive Species Database, IUCN) and that are competent vectors for several diseases^16^, were used as model species to assess whether climatic niches between native and non-native ranges have changed. *Aedes albopictus*, native to Southeast Asia^17^, is regarded to be one of the fastest spreading invasive species worldwide^18^. Its invasion success has been highly promoted by increasing global trade and tourism. In addition to that, the area with suitable climatic conditions for the species is expected to expand under climate change (e.g. Cunze *et al*.^19^).

The closely related *Ae*. *aegypti*, native to Africa^20,21^, is similarly widespread but has a different history of invasion. While the global spread of *Ae*. *albopictus* took place mainly within the last 30–40 years^22^, the spread of *Ae*. *aegypti* into tropical and subtropical regions outside the African continent took place in conjunction with the increase of slave trade in the 16th and 17th century^21^. Today, *Ae*. *aegypti* is restricted to tropical and subtropical regions in which temperatures remain relatively warm throughout the year. Records from moderate climate zones are still missing or rare and may be the result of the species’ domesticity (feeding, mating, oviposition indoors) (e.g. Gloria-Soria *et al*.^23^).

The aim of this study was to assess whether the climatic niches of these two mosquito species have changed or remained stable (niche conservatism hypothesis) during the invasion process. Particularly regarding their different temporal invasion histories (30–40 years here refers to “short-term”, more than 300 years refers to “long-term”, i.e. time that has passed since the first observations outside their native range were made), we expected niche changes to be more likely for *Ae*. *aegypti* than for *Ae*. *albopictus*. The niches of these two closely related species were compared within a single continent (or range) where both species occur and were investigated for their overlap, but also for their similarity and equivalence. Following the applied niche comparison framework for native and non-native ranges by Guisan *et al*.^24^, three basic components were distinguished: niche unfilling, niche stability and niche expansion, accounting for the availability of environmental conditions within the respective range^25^.

## Results

**Figure 1:**
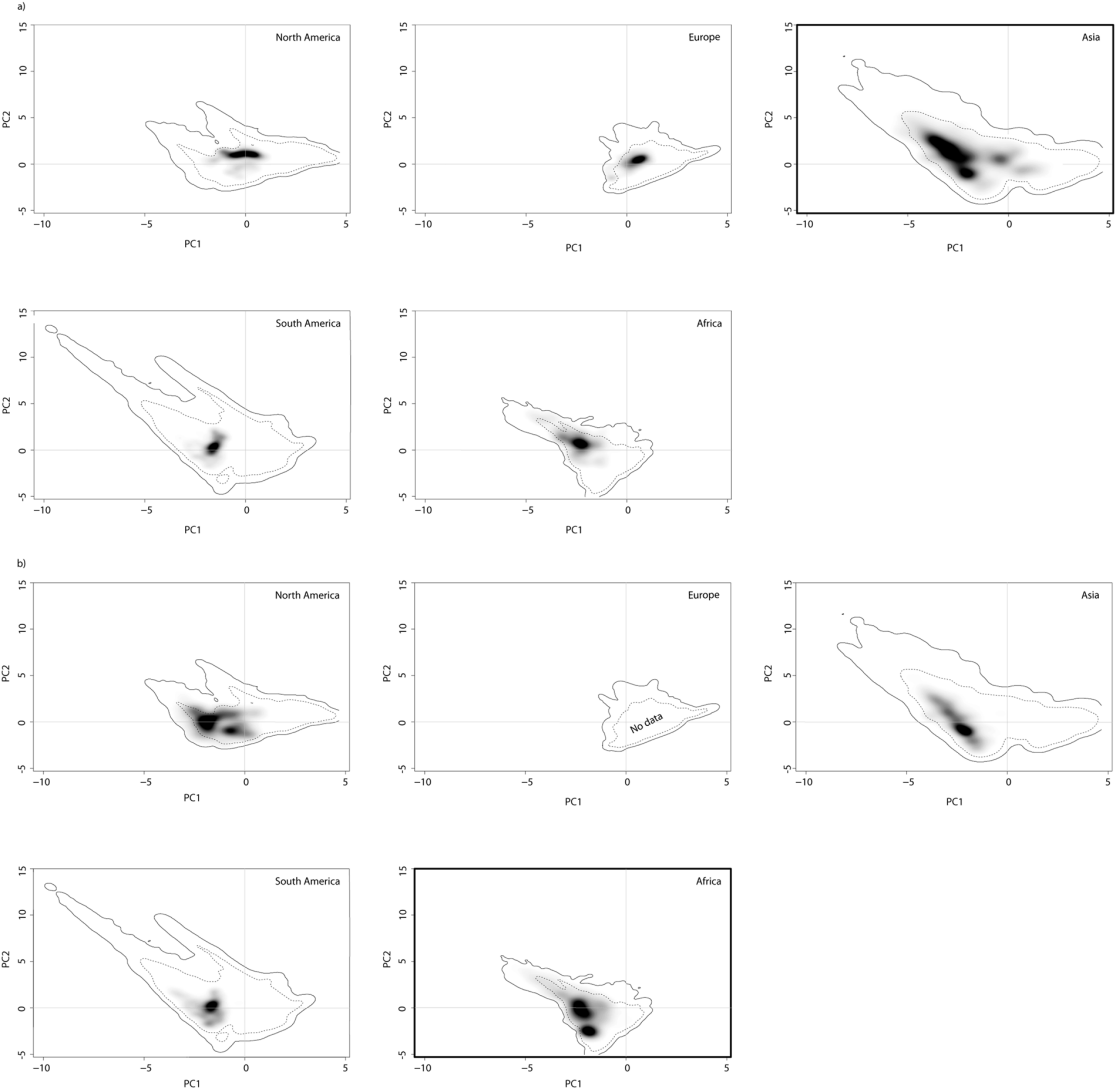
Climatic niches of *Aedes albopictus* and *Aedes aegypti* in the niche space spanned by two PCA-axes. The environment available and the environment occupied (realized niche) by (**a**) *Aedes albopictus* and (**b**) *Aedes aegypti* in the non-native ranges and in the native range (bold frame) are shown. Grey shading represents the density of the occurrences by cell, with darker shading indicating higher density of occurrences. Solid contour lines visualize 100% of the available environment; dashed contour lines indicate 50% of the most common background environment. Figures built using R Package ‘ecospat’ version 2.1.1^43,44^ (www.unil.ch/ecospat/home/menuguid/ecospat-resources/tools.html).

**Figure 2:**
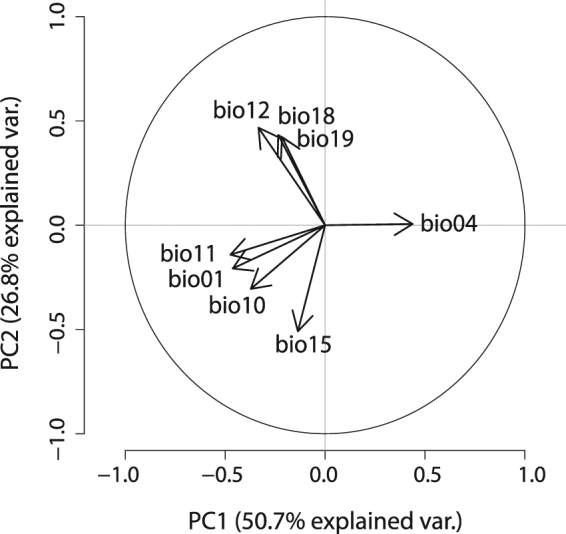
Principle component analysis (PCA) plot. The contribution of the eight bioclimatic variables - Annual Mean Temperature (bio01), Temperature Seasonality (bio04), Mean Temperature of Warmest Quarter (bio10), Mean Temperature of Coldest Quarter (bio11), Annual Precipitation (bio12), Precipitation Seasonality (bio15), Precipitation of Warmest Quarter (bio18) and Precipitation of Coldest Quarter (bio19) - on the two PCA-axes PC1 and PC2 and the percentage of variability explained by these axes are shown in the PCA-plot.

The different shapes of climatic niche space indicate some variation in climate backgrounds, i.e. the available environments, across the considered five geographical ranges (solid and dashed contour lines, Fig. 1). Furthermore, the occupied native and non-native range niches (dark shaded areas) of both mosquito species position differently along the two PCA axes. The first two PCA axes explain 77.5% of the variation in the data. The first PCA axis is positively related to temperature seasonality (bio04) and negatively related to mean temperature in the coldest quarter and to the annual mean temperature (bio11, bio01), whereas the second axis is positively related to the precipitation variables (Fig. 2).

### Niches of *Aedes albopictus*

Climatic niche occupancy of *Ae*. *albopictus* in the Asian native range exceeds the climatic niche occupancy in the non-native ranges; this pattern is evident for all considered non-native ranges, implicating a niche unfilling (Fig. 1a). In comparison to the Asian native range a rightward shift of the niche centroid can be observed, with the highest occupancy of *Ae*. *albopictus* under comparably lower temperatures within the non-native ranges (PC1 axis is negatively correlated to mean temperature in the coldest quarter and to the annual mean temperature), especially in Europe and North America.

In general, the native range niche for *Ae*. *albopictus* shows only little overlap with the non-native range niches (Table 1, Schoener’s D between 0.03 (minimum) for the comparison with the European niche and 0.16 (maximum) for the comparison with the African niche). The niche overlaps between the non-native range niches cover a broader range (Table 1, Schoener’s D between 0 for the overlap of the European and African niches and 0.36 for the overlap of the North and South American niches). For all pairs of ranges except for the pair North America and Europe, the niches of the respective ranges are significantly not equivalent (significance level α = 5%). According to the niche similarity test the North American and the European niche for *Ae*. *albopictus* are more similar than expected by chance (Table 1). The same is true for the Asian compared to the North American niche and the South American compared to the African niche but not vice versa (Table 1).

**Table 1:** Comparison of the native and non-native niches for *Aedes albopictus* and *Aedes aegypti*. Niche overlap was calculated as Schoener’s D comparing the niches in range 1 (R1) and range 2 (R2).

Range 1	Range 2	Niche overlap (D)	Niche similarity		Niche equivalency
			R1 → R2	R2 → R1	
***Aedes albopictus***					
Asia†	Europe	0.03	ns	ns	different*
Asia†	Africa	0.16	ns	ns	different*
Asia†	North America	0.09	similar*	ns	different*
Asia†	South America	0.11	ns	ns	different*
Europe	Africa	0	ns	ns	different*
Europe	North America	0.25	similar*	similar*	ns
Europe	South America	0.02	ns	ns	different*
Africa	North America	0.01	ns	ns	different*
Africa	South America	0.16	ns	similar*	different*
North America	South America	0.36	ns	ns	different*
***Aedes aegypti***					
Africa†	Asia	0.27	similar*	similar*	different*
Africa†	North America	0.18	ns	ns	ns
Africa†	South America	0.18	ns	ns	ns
Asia	North America	0.31	similar*	similar*	different*
Asia	South America	0.28	similar*	similar*	different*
North America	South America	0.45	ns	ns	ns

^†^Native range.

ns = not significant.

^*^The ecological niches are significantly (α = 5%) more similar (similarity test) or different (equivalency test) than expected by chance.

Compared to the native range niche, no niche expansion but a full niche stability can be observed in the invaded ranges (niche expansion = 0, niche stability = 1, Table 2). Niche unfilling compared to the native range niche is relatively high for *Ae*. *albopictus*, ranging from 23% for the African niche up to 87% for the European niche (50.5% on average, Table 2).

**Table 2:** Niche metrics. Comparison of the native ranges and the non-native ranges for *Aedes albopictus* and *Aedes aegypti*.

Species (native range)	Non-native range	Expansion	Stability	Unfilling
*Aedes albopictus* (Asia)	Africa	0.00	1.00	0.23
	Europe	0.00	1.00	0.87
	North America	0.00	1.00	0.55
	South America	0.00	1.00	0.37
*Aedes aegypti* (Africa)	Asia	0.04	0.96	0.00
	North America	0.07	0.93	0.05
	South America	0.00	1.00	0.10

### Niches of *Aedes aegypti*

The comparison of the non-native niches of *Ae*. *aegypti* in South America and Asia with its native range niche in Africa revealed no clear pattern of niche unfilling, expansion or niche shift (Fig. 1). However, in comparison to the African native range, the niche centroid seems slightly shifted to the right in North America towards comparably lower temperatures.

The native niche for *Ae*. *aegypti* shows a slight overlap with the non-native niches (Table 1, Schoener’s D between 0.18 for the comparison with the North and South America niche and 0.27 for the comparison with the Asian niche). The overlaps between the non-native range niches vary between 0.28 and 0.45 (Table 1). The non-native Asian range niche is significantly not equivalent to the native range niche in Africa (Table 1, α = 5%). According to the niche similarity test, the African native and the Asian niche, the Asian and the North American niche as well as the North American and the South American niche for *Ae*. *aegypti* are more similar to each other than expected by chance (Table 1). The Asian niche is more similar to the South American niche of *Ae*. *aegypti* than would be expected by chance, but not vice versa (Table 1).

The comparison of the African native range niche with the non-native range niches reveals a high niche stability (96.33%, averaged over three non-native ranges), a small niche expansion (3.67%), and a very small percentage of niche unfilling (0.05%) in the non-native ranges for *Ae*. *aegypti* (Table 2).

### Comparison of niches between *Aedes albopictus* and *Aedes aegypti* within a single range

In contrast to the niche overlaps between ranges and within single species, a comparison of the niches of both mosquitos within the same range generally reveals higher niche overlaps (higher Schoener’s D values, see Table 3), with the lowest D value for the overlap of the niches of *Ae*. *albopictus* and *Ae*. *aegypti* in North America. In all considered ranges the niches of the two species are not equivalent and seem to be more similar than expected by chance only in Asia (Table 3). Within the considered climatic niche space, the niches of species in their respective native regions exceed the niches of the non-native species in that region, i.e. the niche of *Ae*. *aegypti* exceeds the niche of *Ae*. *albopictus* in Africa by 20% whereas the niche of *Ae*. *albopictus* exceeds the niche of *Ae*. *aegypti* in Asia by 7%.

**Table 3:** Comparisons of *Aedes albopictus* and *Aedes aegypti* niches within a single range.

Range	Niche overlap (D)	Niche similarity		Equivalency
		*Ae*. *albopictus* → *Ae*. *aegypti*	*Ae*. *aegypti* → *Ae*. *albopictus*	
Asia	0.61	similar*	similar*	different*
Africa	0.51	ns	ns	different*
North America	0.19	ns	ns	different*
South America	0.62	ns	ns	different*

ns = not significant.

*The ecological niches are significantly (α = 5%) more similar (similarity test) or different (equivalency test) than expected by random.

## Discussion

The invasion of non-native species is considered a continuous process and sometimes happens within short time spans^12,24,26^, which can challenge a clear designation to the processes involved. According to Guisan *et al*.^24^ differences in realized niches between native and non-native ranges can be ascribed to either a) adaptive evolutionary changes in the physiological tolerance of the species during the invasion process, b) a broad fundamental niche and thus, preadaptation to conditions not available (anymore) within the native range but available within a non-native range, c) changes in biotic interaction and/or d) limitations in dispersal ability of the species. Here, we investigated whether the niches of the two competent vector mosquito species *Ae*. *albopictus* and *Ae*. *aegypti*, involving either a global short- or long-term invasion history, respectively, remained stable (niche conservatism) during the invasion process. In the following paragraphs, we discuss our results focusing more specifically on the different scenarios of niche expansion and niche unfilling for the two species. Possible interpretative approaches of the considered niche parameters and their likelihood are summarized for both species in Table 4.

**Table 4:** Interpretative approach of niche scenarios for *Ae*. *albopictus* and *Ae*. *aegypti*.

Indication	Mechanism		*Aedes albopictus*	*Aedes aegypti*
niche expansion	evolutionary changes		unlikely (no observed expansion)	unlikely (no observed expansion)
	changes in biotic interaction	absence of neg. interaction/presence of new pos. interaction	unlikely	unlikely
niche unfilling	dispersal limitation		likely (short time since invasion started but fast spreading velocity assumed)	unlikely (invasion process started long time ago)
	changes in biotic interaction	absence of pos. interaction/presence of new neg. interaction	unlikely	unlikely

*Aedes albopictus* is a recent global invader and it has been suggested that evolutionary changes through local adaptation might be possible, evidenced by changes in expression of diapause in different studies^27–30^. In concordance with the results of the study by Medley^27^, we also found that the niches of *Ae*. *albopictus* were not equivalent between native and non-native ranges. However, unlike Medley^27^, who assumed a niche change but did not differentiate between the different scenarios of niche changes, we found no evidence for a niche expansion in the non-native range. Rather, we found 100% niche stability for all considered non-native ranges compared to the Asian native range, which confirms niche conservatism according to Guisan *et al*.^24^.

Asia covers a broad range of environments, ranging from tropical to temperate conditions. *Aedes albopictus*, native to the Asian continent, occupies several of these environments and is therefore considered to have a broad native range niche (possibly due to two populations, one occurring under tropical conditions, and one occurring under more temperate conditions, see also discussion further below). The non-native ranges (except for South America) cover smaller ranges of environmental conditions with consequently smaller niches. An obvious shift in the centroids of occupancies compared to the native range niche can be seen towards more temperate conditions (North America and Europe), and towards more tropical conditions (South America and Africa) (Fig. 1). This corroborates the results of Kotsakiozi *et al*.^31^, who recently detected two major genetically differentiated population clusters in the native range.

As well as observing non-equivalent niches of *Ae*. *albopictus*, we found high values of niche unfilling. Generally, high values of niche unfilling indicate an imbalance between species occupied range (i.e. actual distribution) and potential range (i.e. all areas with suitable habitat conditions) and can be interpreted as an indication of the incomplete invasion process. For example and as indicated in Table 4, despite suitable habitat conditions, unoccupied parts might occur in the non-native range, particularly if the invasion process extends over only a short period of time and the species is not in equilibrium with its environment yet. This unfilling of the geographical space could then be reflected in an unfilling of the niche space.

Niche unfilling could, however, also arise from an altered situation of biotic interactions, e.g. the absence of an important symbiosis partners or the occurrence of predators or high competitive pressure in the non-native range. *Aedes albopictus* is considered a strong competitor^23^ (as is *Ae*. *aegypti*), showing a low specialization in its food spectrum as well as a broad spectrum of hosts for blood meal. The most relevant biotic interaction considering these two species would be interspecific competition. Although both species can interact with each other, the outcome seems to be population-specific and rapidly evolving^32,33^. On the other hand, small-scale spatial partitioning might lead to local co-existence of both species^34,35^.

Based on our results, there is no clear indication for a niche expansion that would require evolutionary adaptations of the species during its short-term invasion progress. However, more local adaptive evolutionary changes might still occur and have been observed such as those of photoperiodic response^28,36^ or resistance to satyrization^33^. These studies show evidence for trait evolution and not necessarily for niche evolution compared to native populations. With regard to *Ae*. *albopictus*, two “density-clouds” (depicted in the third graph of the top row in Fig. 1), associated to the more “tropical” or “temperate” populations in the native range in Asia, can be identified. If a “tropical” population was introduced and did subsequently evolve in the US, now showing signs of photoperiodic diapausing, this would not appear as a niche expansion in this approach as the ecological niche of the species would still fall into the extent of the native range niche.

Our results contradict a very recent study by Hill *et al*.^37^ who detected a niche expansion (0.28) and a lower unfilling (0.07) for *Ae*. *albopictus*. These differences might be ascribed to the different study designs, including differences in occurrence data and environmental backgrounds. Whereas we investigated the non-native range niches for each continent separately, Hill *et al*.^37^ considered a global non-native range niche, comprising North America, South America, Europe and Africa. Pooling our data of the non-native range niches we found similar values of low unfilling. Differences in the expansion index seem to underlie more complexity, but might be related to differences in background selection. Whereas we chose continent-wide ranges based on roughly the same number of pixels, ranges chosen by Hill *et al*.^37^ focused on species’ occurrence points within biomes. This more restrictive and narrow background might explain the higher expansion index. Valuable statements about niche expansion are only possible when analog environments are considered in both native and non-native ranges and studies that restricted their analyses to analog environments found niche conservatism to be dominant among invasive species^24^.

*Aedes aegypti* started its global expansion much earlier and consequently had much more time to invade suitable habitats; an equilibrium between potentially suitable niche space and occupied niche space was therefore expected. This was confirmed in our analysis with lower values of niche unfilling compared to *Ae*. *albopictus*. Thus, *Ae*. *aegypti* has been able to occupy its niche in the non-native range over the last four centuries, colonizing all continents except areas beyond 45° latitude including Europe. Furthermore, *Ae*. *aegypti* showed very high niche stability and a very low expansion with more pairs of similar or equivalent niches (native versus non-native niche). We would argue that also with this species, there is no indication for a violation of the assumption of niche conservatism.

Similarly to *Ae*. *albopictus*, *Ae*. *aegypti* has a wide geographical distribution and establishes populations in a variety of environments. The high values of overlap between the niches of both species’ niche in Asia, South America and Africa would suggest a high ecological similarity, however, the niches are not equivalent and only similar to each other in Asia. These differences as well as differences in the geographical ranges (*Ae*. *aegypti* does not occur or only rarely in more temperate areas) become apparent when considering eco-physiological adaptations of each mosquito^38^. *Aedes albopictus* is able to undergo egg diapause (in particular populations from temperate regions) allowing the species to persist during cold temperature periods that are unfavourable to adult survival. *Aedes aegypti* does not have this ability and thus, shows only limited adaptation to egg stage survival in unfavourable periods^38^. Furthermore, the two species have been found to segregate based on land use and human impacts on habitats, thus, *Ae*. *albopictus* tends to be associated with vegetated areas, with more suburban/rural land use, whereas *A*. *aegypti* tends to be associated with urban areas, with high human population density^34,35,39^.

**Figure 3:**
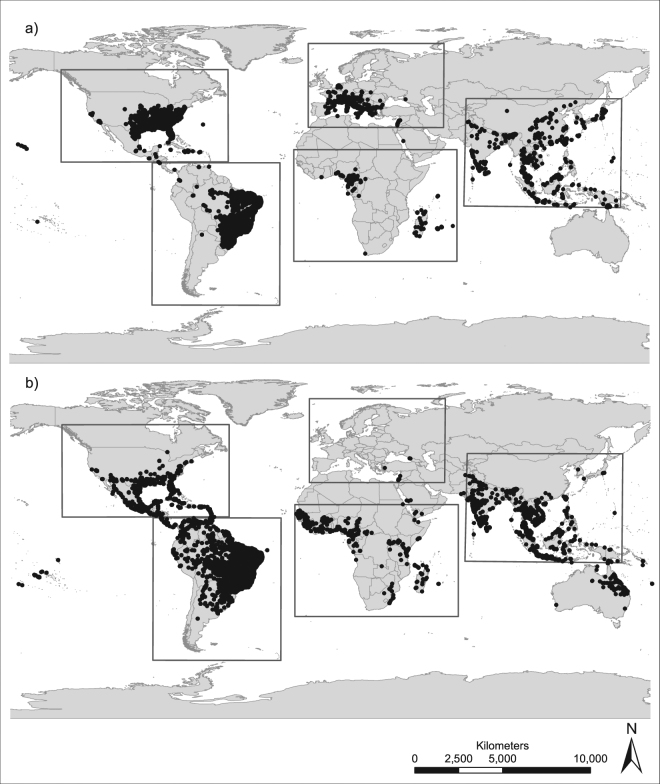
Study areas and occurrence. Records for (**a**) *Aedes albopictus* and (**b**) *Aedes aegypti*. Grey boxes indicate the boundaries of the five considered ranges: North America, South America, Europe, Africa and Asia. Maps were built using ESRI ArcGIS 10.3^53^ (www.esri.com/software/arcgis).

The ability to undergo a diapause in the egg stage, a generally common characteristic of insects^40^, is largely considered an adaptation to temperate climatic conditions. Due to the wide latitudinal distribution in the ancestral native range in Asia, two distinct strains are proposed for *Ae*. *albopictus*: a tropical one that does not have to ability to undergo photoperiodic diapause when conditions are unfavourable, and a temperate one with a phenotypically plastic diapause response (see review by Armbruster^28^). It could be speculated that the pattern of the native Asian range niche of *Ae*. *albopictus*, with the two occurrence “density-clouds”, would be an indication for this theory. Furthermore, it seems that the North American and European niches match the more temperate occurrence density hotspot (near the point of origin in the considered niche space, Fig. 3). Strong evidence that the invasion history of North America was from a temperate origin has formerly been proposed by Armbruster^28^ and was recently confirmed using population genomics^31^. The South American and African niches are more similar in environmental conditions to the tropical occurrence density hotspot in the native range. Finally, assessing evolutionary niche changes during invasion relies on the knowledge of fundamental niches in the native and non-native ranges, which is very difficult to obtain and only exists for very few species. Future research of different invasive and native populations and their diapausing response in relation to latitude, also including reciprocal experiments, is still needed to further investigate differences between the aforementioned populations/strains (but see first attempts)^29,30,41,42^.

Considering the occurrence points used in this study (see Material and Methods section below), it was not possible to verify whether mosquito populations were established in each location. Thus, there could be locations that are re-colonized each year but don’t persist without immigration. Assuming that the mosquito species have not become established in some of these locations and the occurrence records have been just single findings, these records would fall outside their realized niche, i.e. the non-native niche would have been overestimated. However, as we did not find signs of a climatic niche expansion, wrongly taking these records into account does not change the main conclusion of our results. We consider our approach to be conservative and appropriate, especially for precautionary risk assessments or management of invasive species.

Due to current constraints on characterizations of the fundamental niche, most studies focus on realized niches (derived from distributional data) and apply metrics of niche unfilling, niche stability and niche expansion, while accounting for availability of environmental conditions in the respective ranges^24^. Applying an approach developed by Broennimann *et al*.^25^ implemented in the “ecospat” package^43,44^, we here confirmed the theory of niche conservatism for *Ae*. *albopictus* and *Ae*. *aegypti* without any indications for a niche expansion during their global route of invasion. For *Ae*. *albopictus*, a niche unfilling was shown, which explains the present differences between native and non-native range niches. The environmental space currently unoccupied by the species in the non-native ranges relates most likely to the short time span since the global spread of the species (Table 4). On the contrary, it appears that *Ae*. *aegypti* has been able to occupy its native niche in the non-native range over the last four centuries, colonizing all continents except areas beyond 45° latitude including Europe. In conclusion, differences in niche unfilling suggest two different stages of invasion for the two species; whereas *Ae*. *aegypti* might already be in equilibrium with the environment, this is not the case for *Ae*. *albopictus*. We would support the use of native-range based niche models to be used for projections of areas at a risk of invasion in the future, and confirm climatic niche conservatism as a valid underlying assumption, which does not exclude the potential of the species for evolutionary adaptations on a local scale.

## Material and Methods

### Data specifications and niche approach

We used occurrence records of both species from Kraemer *et al*.^45,46^. The coordinates of the records were adjusted to the raster of the environmental variables with a spatial resolution of 5 arc minutes (~10 km × 10 km) and accounted for only one occurrence record per grid cell in order to minimize spatial autocorrelation. The numbers of occurrences after adjusting to the 5 arc minutes resolution are given in Table 5. We considered five biogeographically separated regions with observed distribution for *Ae*. *albopictus* and *Ae*. *aegypti*: Asia (native range of *Ae*. *albopictus*), Africa (native range of *Ae*. *aegypti*), Europe, North America and South America (see Fig. 1). We chose the extents of these study areas so that they – if possible – cover all records in this region, and comprise approximately the same number of pixels. Despite the occurrence of *Ae*. *aegypti* in Australia, we decided not to consider Oceania in our study to keep the number of pixels for backgrounds equal for all considered continents. Established populations of *Ae*. *albopictus* were found in 2005 in Australia (on islands in the Torres Strait, between mainland Australia and Papua New Guinea). However, it was shown that due to effective control programmes, an expansion of *Ae*. *albopictus* was successfully prevented^47^. We would therefore expect a niche unfilling evoked by an anthropogenic dispersal limitation for *Ae*. *albopictus* in Australia. For *Ae*. *aegypti* the European range was not taken into consideration due to the small number of recorded occurrences in Europe (probably due to unsuitable climate). Eight out of 19 available bioclimatic variables provided by worldclim^48^ were selected to cover climatic variables that are considered ecologically relevant for both species. Those variables included annual mean, amplitude and seasonal variation for both temperature and precipitation, specifically: Annual Mean Temperature (bio01), Temperature Seasonality (bio04), Mean Temperature of Warmest Quarter (bio10), Mean Temperature of Coldest Quarter (bio11), Annual Precipitation (bio12), Precipitation Seasonality (bio15), Precipitation of Warmest Quarter (bio18) and Precipitation of Coldest Quarter (bio19). These eight bioclimatic variables are inter-correlated, but were transformed into non-correlated linear combinations (principal components) of the original variables^45^ by a principle component analysis (PCA). The first principal component accounts for as much of the variability in the data as possible, each succeeding component accounts for as much of the remaining variability as possible. To characterize the climatic niches of the two mosquito species, we applied the principal components analysis PCA-env approach developed by Broennimann *et al*.^25^. This approach works in the two-dimensional gridded environmental space (100 × 100 grid) spanned by the first two axes, which were derived from the PCA based on the above mentioned eight bioclimatic variables considering all five ranges together.

**Table 5:** Number of occurrence points after adjusting to the 5 arc minutes resolution and extents of the considered ranges (N – latitude of the northern margin, E – longitude of the eastern margin, S – latitude of the southern margin and W – longitude of the western margin of the bounding box).

	*Aedes albopictus*	*Aedes aegypti*	N	E	S	W
North America	1453	600	60.50°N	−137.50°E	13.17°N	−52.58°E
South America	3324	4962	12.75°N	−90.25°E	−59.42°N	−26.17°E
Europe	356	(6)*	76.50°N	−15.00°E	29.58°N	57.67°E
Africa	806	2502	19.42°N	−17.50°E	−37.42°N	63.50°E
Asia	4204	6471	45.83°N	70.00°E	−11.08°N	149.33°E

^*^Not included in the analysis.

### Niche metrics

The niches were visualized and compared between native and non-native ranges accounting for niche overlap, niche similarity, niche equivalency, niche stability, niche expansion and niche unfilling, for *Ae*. *albopictus* and *Ae*. *aegypti*, respectively. In addition, we compared the niches of *Ae*. *albopictus* and *Ae*. *aegypti* within each of the four considered ranges where both species occur. The different niche metrics are described in the following paragraph.

The niche overlap corresponds to the intersection of two niches in the niche space. We considered Schoener’s D^49,50^, the most commonly used measure for niche overlap. Schoener’s D is an index ranging between 0 and 1, where 0 means no overlap between the considered niches and 1 means a total overlap between the considered niches, i.e. the two niches are identical.

In addition, we applied the niche equivalency test and the niche similarity test^50^. These are two frequently used statistical tests considering hypotheses of niche divergence or conservatism^51^. The niche equivalency test determines whether the niche overlap is constant when randomly reallocating the occurrences of the species among the two ranges^25^. More specifically, the equivalence test compares the overlap of both niches with the overlap of simulated niches; it pools the occurrences of both ranges and randomly splits the pooled data into two datasets, maintaining the number of occurrences as in the original datasets. On the other hand, the niche similarity test addresses whether the overlap between ecological niches in two ranges is different from the overlap between the niche in one range and niches selected at random from the other range^25^. The niche equivalency test is conservative as it only tests if the modelled niches of the two species are identical in their niche spaces accounting for the occurrences only and not for the background^14,50^. The niche similarity test assesses if the considered niches are more similar than expected by chance, accounting for the differences in the surrounding environmental conditions in the geographic areas where both species are distributed^14,53^. Significant differences according to the niche equivalency test together with significant similarity according to the niche similarity test means that niches are similar, but not equivalent.

As we aimed to test for niche conservatism we used the one-tailed test (alternative = “greater”, in the settings) and tested if niches are more similar (similarity test) or different (equivalency test) than expected by chance. For both tests we used 1000 permutations to evaluate the significance (α = 5%) of niche equivalency and niche similarity.

Furthermore, we evaluated niche stability (i.e. the proportion of the non-native range niche overlapping with the native range niche), niche expansion (i.e. the proportion of the non-native range niche not overlapping with the native range niche) and niche unfilling (proportion of the native range niche not occupied in the non-native range niche).

Niche expansion and niche stability always add up to 100%. The niche unfilling value would correspond to the niche expansion value when switching native range and non-native ranges.

Niche conservatism is defined as the tendency for species to retain their niche in space and time and is used synonymously with ‘niche stability’^24^. Niche conservatism is given when the niche stability is 100% and niche expansion is 0% - regardless of the niche unfilling.

All analyses were performed in R environment version 3.3.1^52^. Niche comparisons were performed and visualized using “ecospat” package^43,44^.
